# Aggression and cortisol levels in three different group housing routines for lactating sows

**DOI:** 10.1186/s13028-015-0101-7

**Published:** 2015-02-18

**Authors:** Ola Thomsson, Ann-Sofi Bergqvist, Ylva Sjunnesson, Lena Eliasson-Selling, Nils Lundeheim, Ulf Magnusson

**Affiliations:** Division of Reproduction, Department of Clinical Sciences, Swedish University of Agricultural Sciences, SLU, Box 7054, SE-750 07, Uppsala, Sweden; Swedish Animal Health Service, Kungsängens Gård, SE-753 23, Uppsala, Sweden; Department of Animal Breeding and Genetics, Swedish University of Agricultural Sciences, SLU, Box 7023, 750 07, Uppsala, Sweden

**Keywords:** Organic production, Swine, Suckling, Stress, Agonistic behaviour, Cortisol, Housing

## Abstract

**Background:**

Lactating sows in Swedish organic piglet production are commonly group-housed with piglets in a multi-suckling pen within 14 days after farrowing. Nursing behaviour may be disturbed when lactating sows are moved to a new environment and mixed with other sows, as they spend more time fighting with other sows and exploring the new surroundings. This can disrupt the inhibitory effect of suckling on ovarian activity and increase the risk of lactational oestrus, making efficient reproductive management difficult. Therefore this study evaluated aggression and levels of the stress hormone cortisol in lactating sows group-housed together with their piglets at one (W1), two (W2) or three (W3) weeks post farrowing.

**Results:**

There was no significant difference (*P* > 0.05) between the three management routines (W1, W2, W3) regarding number of attacks initiated or received in the mixed group. After mixing, W2 sows had a lower number of shoulder scratches (*P* < 0.05) than W3 sows. Among the W3 sows, there was a lower (*P* < 0.01) cortisol concentration in saliva when sows were group housed compared to when they were individually housed. The cortisol response, measured as variation in cortisol concentration in saliva, was also lower (*P* < 0.05) in group-housed W3 sows compared with W1 sows.

For all management routines, sows already living in the new environment (resident sows) initiated more attacks (*P* < 0.001) and received fewer attacks (*P* < 0.01) than sows entering the new environment (intruder sows). Overall, multiparous sows initiated more attacks and received fewer attacks than primiparous sows (*P* <0.001).

**Conclusions:**

Overall, the results suggest that mixing and group housing sows at three weeks post farrowing is less stressful than mixing and group housing sows at one week post farrowing. The results also indicate that parity and whether a sow is a resident or intruder in the group housing environment may have an effect on aggression levels when sows are group-housed.

## Background

The efficiency of Swedish organic piglet production is negatively affected by the high occurrence of lactation oestrus [1,2] and high piglet mortality [3]. In this form of production, the sows farrow in individual farrowing pens and are commonly moved within 14 days after farrowing to a multi-suckling pen, where batches of lactating sows and piglets of similar age are housed together and provided with outdoor access [4,5]. The farrowing dates within a batch of sows are spread out over time and the move to a multi-suckling pen often involves sets of sows that have farrowed most closely in time. In the multi-suckling pen, the sow has a higher chance of escaping the piglets, implying that suckling is less frequent for some sows and does not exert the same inhibitory effect on ovarian activity as when the sow and piglets are housed in an individual farrowing pen throughout lactation [6]. Hence the reduced suckling frequency in the multi-suckling pen could contribute to the occurrence of lactational oestrus within organic piglet production. Lactational oestrus disrupts the batch-wise breeding regime and results in a prolonged weaning-to-service interval at herd level, with fewer piglets produced per sow and year [3,7,8].

It has been reported that nursing behaviour is disrupted when lactating sows are moved to a new environment and mixed with other sows, as they spend more time fighting with the other sows and exploring the new surroundings [9]. These events are known to be stressful and stress can be assessed by measuring cortisol in blood or saliva [10-12]. Given the critical role of nursing-suckling interaction in maintaining lactational anoestrous in the sow, the aim of this study was to investigate the effect of different group-housing and mixing routines on aggression and stress.

## Methods

### Animals and experimental design

The study was conducted in the period 7 September-14 November 2011 at the Swedish University of Agricultural Sciences Research Station at Funbo Lövsta, just outside the city of Uppsala. Three multi-suckling pens were constructed in an uninsulated barn. Mean outdoor temperature during the study decreased from 13°C in September to 5°C in November [13]. The study was approved by the Uppsala Animal Ethical Committee (C154/11).

**Figure 1:**
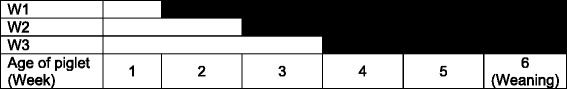
**Illustration of the three management routines, in which sows and their piglets were mixed 1, 2 and 3 weeks post farrowing (W1, W2 and W3).** Black indicates time spent group-housed and white indicates time spent individually housed.

A total of 43 Yorkshire sows were used in the study, of which 11 were first parity sows, 15 second parity sows and 17 sows in parity three to nine. Mean weight at farrowing was 209 ± 14 kg for the primiparous sows and 284 ± 29 kg for the multiparous sows. Mean number of piglets per sow at moving from the individual farrowing pen to the multi-suckling pen was 10 (range 5–15 per sow). One week before expected farrowing, all sows and gilts were moved from a gestation pen where they had been group-housed to conventional individual farrowing pens (8.2 m^2^), where they were loose-housed. The sows were then assigned to one of three different management routines (Figure 1). These routines differed in terms of the number of weeks the sow and her piglets spent in the individual farrowing pen post-farrowing, which was one (W1), two (W2) or three (W3) weeks before they were transferred to the multi-suckling pen. In total, 14 sows were included in management routine W1, 13 sows in W2 and 16 sows in W3. The experiment was performed in two replicates, with 5–8 sows in each replicate and management routine. Within management routine and replicate, sows were assigned to two subsets depending on farrowing date. The first subset of sows (3–6 sows) comprised the first sows to be moved into the multi-suckling pen and these are referred to hereafter as resident sows. The second subset of sows (2–5 sows) was moved to the multi-suckling pen one or three days later and these are referred to hereafter as intruder sows. All piglets were weaned at six weeks of age. Thus, the number of weeks spent group-housed also differed between the management routines, with W1 sows spending five weeks group-housed, W2 sows four weeks and W3 three weeks.

Saliva from the sows was collected for cortisol analysis, starting 2.5 days before the sow was moved to the multi-suckling pen and continuing for six consecutive days, i.e. ending 3.5 days after the commencement of group housing. Cortisol in saliva was analysed as a proxy for endocrinological stress response in the sows [14]. Throughout the group-housing period, the sows were filmed using infrared-sensitive cameras to record agonistic behaviour. Shoulder scratches on the sows were also recorded one day before and on one other occasion 2–7 days after mixing.

### Housing conditions

**Figure 2:**
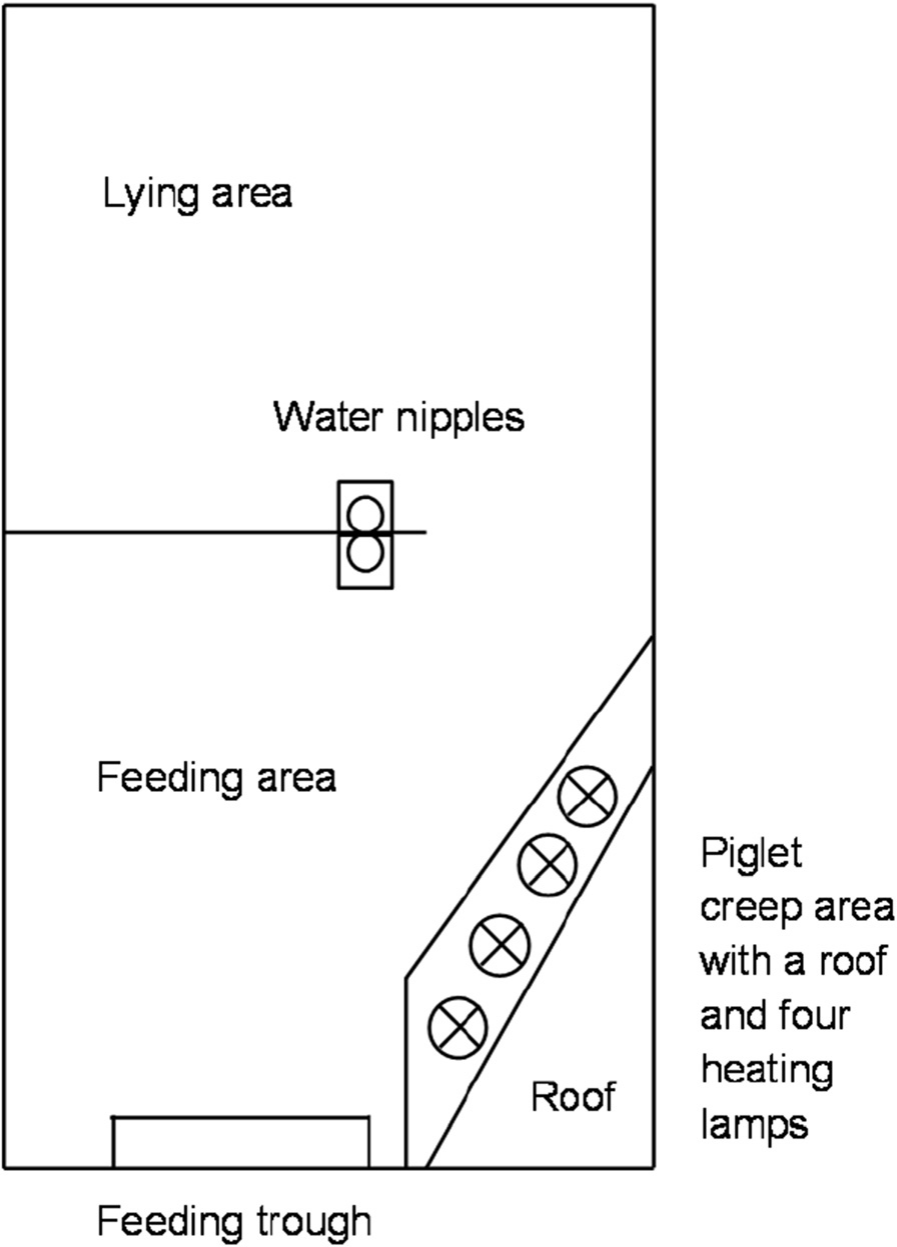
**Illustration of a multi-suckling pen for group-housed sows and piglets.**

The total sow area of each multi-suckling pen was 62.5 m^2^ and the piglet creep area was 5.5 m^2^ (Figure 2). The space allowance per sow met Swedish organic standards and varied between 7.8 and 12.5 m^2^ depending on the total number of sows in the pen [4]. The sow area was divided by a wall into two areas, a feeding area and a lying area. There was a 2.15 m wide gap between the end of this dividing wall and the outer wall of the pen that allowed the sows and piglets to move back and forth between the two sections at all times. The piglet creep area had four heating lamps and a roof about 0.5 m above the floor and was situated in a corner of the feeding area that was inaccessible to the sows. The pen was equipped with two water nipples with bowls underneath to provide the sows and the piglets with water ad libitum. One nipple was located in the lying area and the other in the feeding area. The sows were fed ad libitum with the same dry feed (DIA 120; 12.8 MJ/kg; 160 crude protein/kg, Lantmännen, Sweden) during the entire lactation period. The feeding trough was accessible at all times and the piglets could also eat from the trough. In addition, hay was available ad libitum. Peat and straw were used as bedding material in the feeding area, while only straw was used in the lying area. The sows and the piglets did not have outdoor access from the group-housing pen or the individual farrowing pen. Artificial light was provided between 08.00 and 16.00 h.

### Assessing aggression

Aggression was assessed by studying the video recordings of agonistic behaviour and by counting shoulder scratches. Each multi-suckling pen was equipped with three or four infrared-sensitive cameras that continuously recorded activities in the pen from the time the first sow and her piglets entered the pen until weaning. To allow the sows to be identified in the video, each sow was individually marked on her back with standard colour spray.

Information on agonistic behaviour collected from video recordings included: pushing; fighting; biting; hunting; and threatening, determined according to Kirschner *et al.* [15]. In brief, these behaviours comprised the following: Pushing: a sow being displaced by another sow; Fighting: two sows with body contact in an antiparallel position; Biting: a sow biting or snapping at another sow; Hunting: one sow chasing another sow; Threatening: a sow approaching a sow and pushing her head towards it. Threatening included no body contact and fighting could be accompanied by hunting. If two consecutive actions by the same sow were separated by two seconds, then the actions were recorded as two separate actions. Attack is used hereafter as a generic term for all agonistic behaviour. Video observation was performed in the multi-suckling pen for two continuous hours on four consecutive days, according to Tan *et al.* [16]. The observation starting time for a group was determined as the time the intruder sows had entered the multi-suckling pen and the group was complete. The same starting time was then used for the next three days. The last day of observation corresponded with the last day of saliva sampling of intruder sows.

Shoulder scratches were recorded according to Séguin *et al.* [17]. In brief, an injury penetrating the skin was defined as a scratch. Scratches were counted on both shoulders on the day before and on one occasion 2–7 days after the group housing commenced. Each shoulder was categorised from 0 to 3 based on the number of scratches and the categories were added up to a total score for each sow. This total score was used to determine degree of shoulder scratches, where a score of 0 = none; 1–2 = mild; 3–4 = moderate and 5–6 = multiple.

### Saliva sampling and cortisol analysis

Saliva for cortisol analysis was collected in the morning, starting at 07.00 h, and in the afternoon, starting at 17.00 h. The morning sample on the third day of sampling was the last sample collected in the individual farrowing pen. Thus, the afternoon sample on the third day of sampling was the first sample collected during group housing. This resulted in a total of five samples per sow being collected during the individual housing period and seven samples being collected during the group housing period.

Saliva was collected using a cotton swab (Sarstedt Salivette® for saliva collection ref. 51.1534, Sarstedt, Nümbrecht, Germany) held with forceps. The sow was allowed to chew on the cotton swab until it was saturated, which took 20–60 seconds. The cotton swab was then centrifuged (Hettich Centrifuge EBA 20, Andreas Hettich Group, Ltd., Tuttlingen, Germany) at 2400 x g at room temperature for 2 minutes and the saliva extracted was stored at −20°C before being analysed using a commercially available cortisol ELISA kit (Cortisol ELISA, IBL International, Hamburg, Germany) validated for pig saliva [18].

### Statistical analysis

The statistical analyses were performed using the SAS software ver. 9.3 (SAS Inst. Inc., Cary, NC, USA). In order to describe aggression, the median number of attacks initiated and attacks received across all four days for each sow were used. For analysing the difference between management routines in terms of attacks initiated and attacks received, the Kruskal-Wallis test was used. For differences between resident and intruder sows and between primiparous and multiparous sows, a Wilcoxon sum rank test was used (PROC NPAR1WAY). Shoulder scratches were graded as mean scratch score within management routine and parity (multiparous or primiparous) and differences between management routines were investigated using one-way ANOVA and differences between parities using t-tests.

Data on cortisol concentrations in saliva before and after mixing were analysed in terms of actual concentration and also within-sow coefficient of variation (CV) of the cortisol concentration after mixing. The CV variable was introduced as a means of enabling comparison of the stress-induced cortisol response between sows with different basal cortisol levels and of capturing changes in the diurnal rhythm in the sows [19,20]. The cortisol concentration was not normally distributed and a Wilcoxon rank sum test (PROC NPAR1WAY) was used for calculating differences between individual farrowing pen and multi-suckling pen within management routine and parity (multiparous, primiparous). The cortisol variation was only calculated during group housing and a Shapiro-Wilk test revealed that CV was normally distributed. Analysis of variance (PROC GLM) was used for statistical analysis of CV. The fixed effects included in the statistical model were management routine (W1, W2, W3), resident/intruder and the interaction between management routine and resident/intruder. The effect of parity (primiparous/multiparous) and replicate were included in an initial model, but were found to be non-significant and therefore omitted from the final model. A paired *t*-test was used to compare the cortisol level during individual housing and group housing. For cortisol analyses, data from only 42 sows were available, because one sow refused to chew on the cotton swab. A *P*-value of <0.05 was considered statistically significant.

Spearman correlations between cortisol variation during group housing and agonistic behaviour (broken down to number of attacks initiated and attacks received) were estimated separately for primiparous and multiparous sows. Correlations between scratch score and attacks initiated and attacks received were also calculated.

## Results

### Agonistic behaviour and shoulder scratches

**Figure 3:**
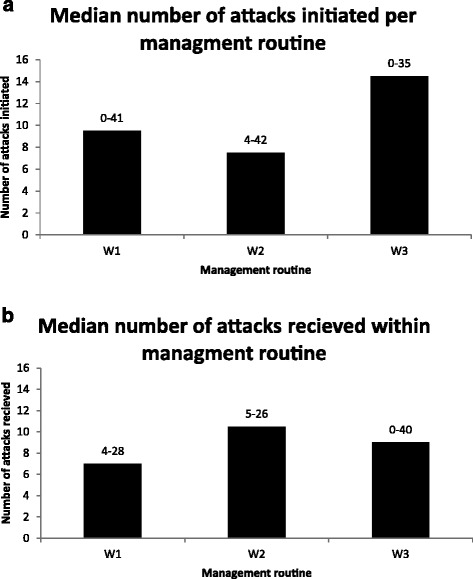
**Median number of (a) attacks initiated and (b) attacks received per sow within management routines W1, W2 and W3, recorded during 2 h for four consecutive days (W1** ***n*** **= 14; W2** ***n*** **= 13; W3** ***n*** **= 16).** The range of number of attacks within management routine is reported above each bar. There were no significant differences between management routines.

**Figure 4:**
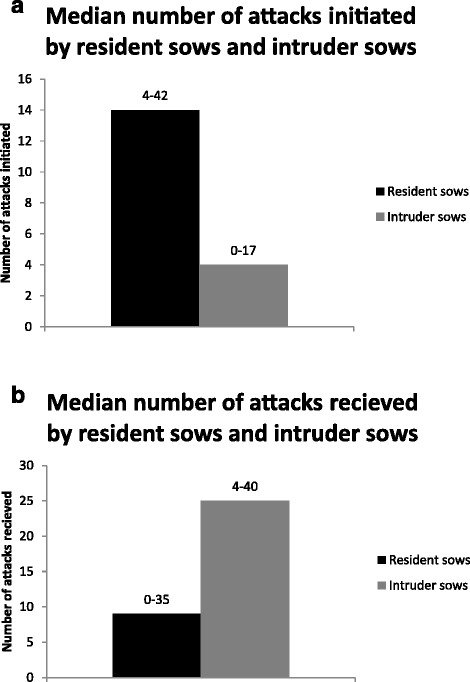
**Median number of (a) attacks initiated and (b) attacks received by resident sows (** ***n*** **= 26) and intruder sows (** ***n*** **= 11), recorded during 2 h for four consecutive days.** Resident sows were moved first to the multi-suckling pen and intruder sows were moved on the following day or three days later. The range of number of attacks within groups of sows is reported above each bar. The two groups differed significantly in terms of both parameters (a: *P* < 0.001; b: *P* < 0.01).

**Figure 5:**
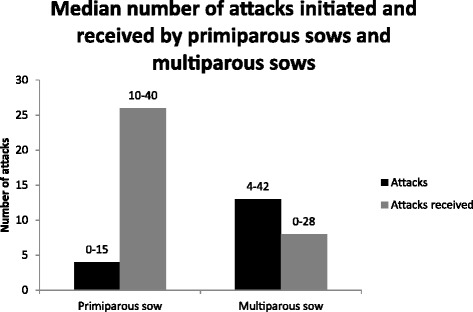
**Median number of attacks initiated and attacks received by primiparous sows (** ***n*** **= 11) and multiparous sows (** ***n*** **= 32), recorded during 2 h for four consecutive days.** The range of number of attacks initiated/received within groups is reported above each bar. There were significant differences between multiparous and primiparous sows for both parameters (*P* < 0.001).

There was no difference between the three management routines regarding the number of attacks initiated or received per sow (Figure 3a, b). For all management routines, resident sows initiated more attacks (*P* < 0.001) and received fewer attacks (*P* < 0.01) than intruder sows (Figure 4a, b). Overall, multiparous sows initiated more attacks and received fewer attacks than primiparous sows (*P* < 0.001) (Figure 5).

**Figure 6:**
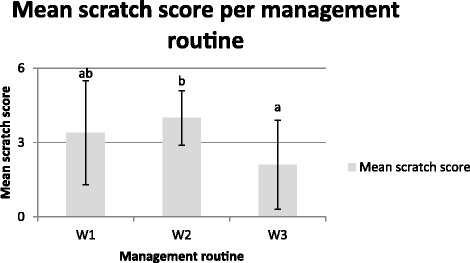
**Scratch score (mean ± S.D) in management routine W1, W2 and W3.** Scratch score: 0 = none; 0 > 3 = mild; 3 > 5 = moderate; ≥5 = multiple. Bars with different letters are significantly different (*P* < 0.05).

**Figure 7:**
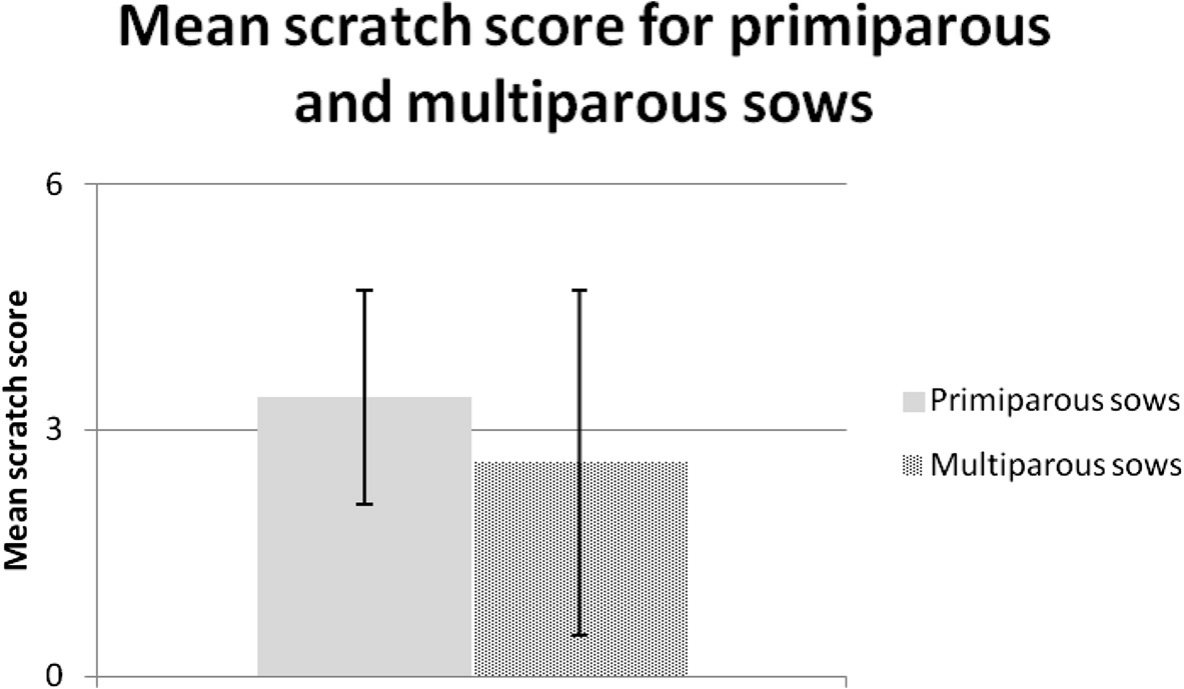
**Scratch score (mean ± S.D) for multiparous sows (** ***n*** **= 32) and primiparous sows (** ***n*** **= 11). Scratch score 0 = none; 0 > 3 = mild; 3 < 5 = moderate; ≥5 = multiple.**

The sows that were group-housed at two weeks post farrowing (W2) had a higher scratch score (*P* < 0.05) than the sows mixed at three weeks post farrowing (W3) and there was a tendency (*P* = 0.06) for a higher score for sows mixed at one week post farrowing (W1) compared with W3 sows (Figure 6). There was no difference in scratch score between primiparous and multiparous sows (Figure 7).

### Cortisol concentrations in saliva when individually and group housed

Sows mixed at three weeks post farrowing (W3) had a higher (*P* = 0.007) cortisol concentration during individual housing compared with group housing (Table 1). There were no such differences between sows that were mixed at one (W1) or two weeks (W2) post farrowing.

**Table 1:** **Cortisol concentration in saliva (mean ± S.D) from sows mixed 1, 2 and 3 weeks post farrowing (W1, W2 and W3) during the time they were individually housed and group housed**

**Management routine**	**Individually housed**	**Group housed**
W1 (*n* = 14)	0.77 ± 0.43 μg/dl	0.54 ± 0.14 μg/dl
W2 (*n* = 13)	0.72 ± 0.36 μg/dl	0.63 ± 0.37 μg/dl
W3 (*n* = 16)	0.82 ± 0.49 μg/dl^a^	0.47 ± 0.24 μg/dl^b^

^ab^Different superscripts within row indicate significant difference (*P* < 0.01).

**Figure 8:**
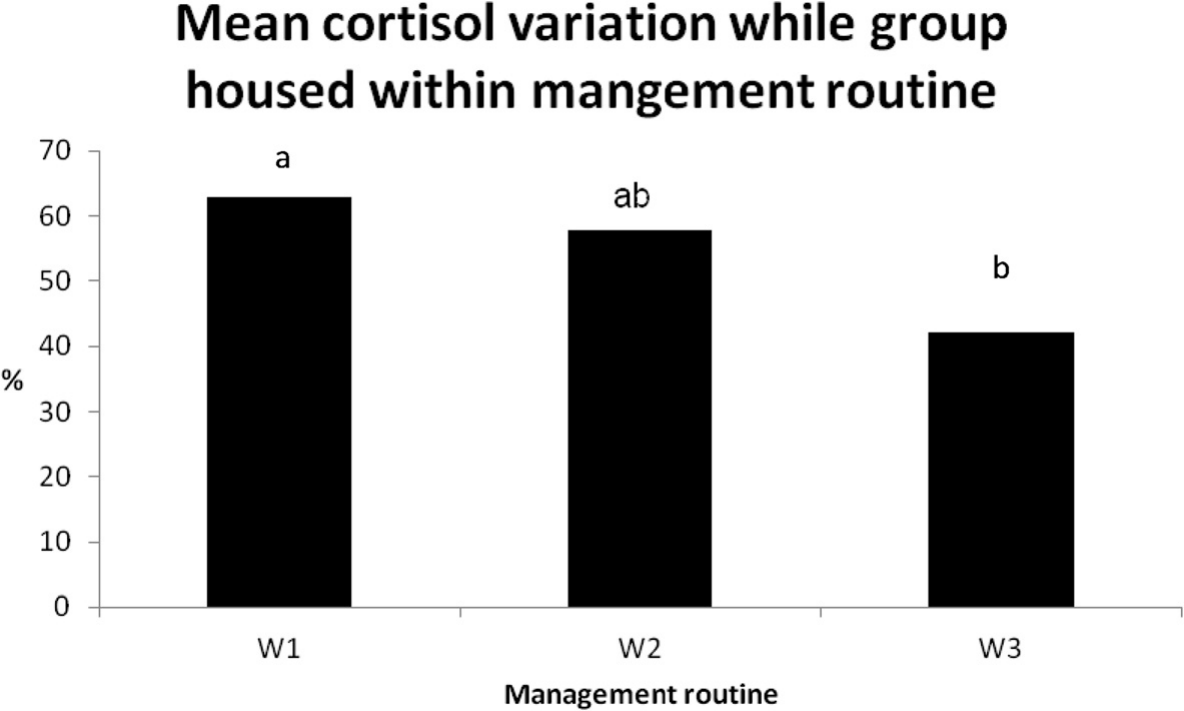
**Mean cortisol variation in sows while group-housed in management routine W1, W2 and W3.** Bars with different letters are significantly different (*P* < 0.05).

The sows in management routine W3 showed a lower CV during group housing compared with W1 sows (*P* = 0.04), but not W2 sows. There was no difference in CV between W1 and W2 sows (Figure 8). Moreover, there was no difference in CV between intruder sows across management routines. However, resident W1 and W2 sows showed higher CV (*P* < 0.01) during group housing than resident sows in W3. Within W3, the resident sows showed a lower CV (*P* = 0.03) compared with intruder sows. There was no difference in CV between housing systems for multiparous and primiparous sows.

### Correlations

For the primiparous sows, there was a negative correlation between cortisol variation during group housing and number of attacks initiated (*P* = 0.004) and between cortisol variation and number of attacks received (*P* = 0.04) (Table 2). For the multiparous sows, there was a tendency for a negative correlation between cortisol variation and attacks received (*P* = 0.09) and a tendency for a positive correlation (*P* = 0.07) between scratch score and attacks received. No significant correlations were found between scratch score and cortisol variation or between scratch score and number of attacks.

**Table 2:** **Spearman correlation coefficient and P-value for aggression traits and cortisol variation in group-housed primiparous and multiparous sows**

	**Primiparous (** ***n*** **= 11)**		**Multiparous (** ***n*** **= 32)**	
**Variables analysed**	**Correlation coefficient**	***P*** **-value**	**Correlation coefficient**	***P*** **-value**
No. of attacks initiated – Cortisol variation while group-housed	−0.82	*0.004*	0.06	*0.79*
No. of attacks received – Cortisol variation while group-housed	−0.65	*0.04*	−0.35	*0.09*
Scratch score – Cortisol variation while group-housed	−0.37	*0.30*	0.12	*0.58*
Scratch score – No. of attacks initiated	0.28	*0.43*	−0.14	*0.47*
Scratch score – No. of attacks received	−0.02	*0.94*	0.36	*0.07*

## Discussion

Differences in aggression and stress (recorded as cortisol level and inter-sow variation) were recorded in sows that were mixed for group housing at different time points after weaning. The differences observed in these traits were also related to whether the sow was an intruder or resident sow at mixing and on whether she was primiparous or multiparous.

Agonistic behaviour did not differ between the three management routines. However, the results indicated that resident lactating sows made more attacks and received fewer attacks than intruding lactating sows. This confirms findings in previous resident-intruder studies [21,22]. Furthermore, primiparous sows, which have a smaller body size than multiparous sows, received more attacks than multiparous sows and initiated fewer attacks. This was possibly due to differences in body size, as this is known to be an important factor for both the direction of aggression (larger sows attacking smaller sows) and rank determination after mixing sows [12,23]. The shoulder scratch data would perhaps have been more significant if a larger area of the body had been used for counting skin lesions. For example, Turner *et al.* [24] found that reciprocal fighting results in more scratches on the anterior third of the body, while fighting of a bullying character results in more scratches on the posterior third of the body.

Interestingly, sows mixed three weeks post farrowing (W3) had a lower basal cortisol concentration in the multi-suckling pen than in the individual farrowing pen, which was not the case for the sows mixed one or two weeks post farrowing (W1 and W2 sows). The difference within W3 agrees with previous findings of reduced cortisol levels when pigs are moved from a barren to an enriched environment [25,26]. In the present study, the multi-suckling pen possessed three characteristics which have been reported to decrease aggression at mixing, namely greater space allowance per sow; ad libitum feeding; and access to bedding material [25-28]. The combination of these three aspects possibly contributed to the lower cortisol level for management routine W3. In addition, the W3 sows could have experienced the farrowing pen as more stressful two days prior to moving due to crowding, as the 3-week-old piglets were heavier than those in the other two management routines [29]. However, it should be noted that a higher cortisol level or no difference in cortisol levels have been reported when mixing pigs, moving pigs from a barren environment (no straw provided) to an enriched environment (straw provided) or solely housing pigs in an enriched environment [30-33].

Sows in management routine W3 had a significantly lower variation in cortisol when group housed compared with W1 sows. This difference could perhaps be related to the lactational stage of the sow. By the second week of lactation, the majority of nursing events are initiated by the piglets and around day 18 post farrowing the sow reaches peak lactation [34,35]. It could be speculated that in group housing, the W1 sows focused more on managing nursing their piglets in the new environment compared with the W3 sows, which had older and more resilient piglets and thus experienced less stress in that regard. The fact that one subset of W3 sows was moved to the multi-suckling pen three days after the first subset, instead of one day, did not significantly affect the CV of the sows (data not shown).

It has been reported that previous experience in pigs of agonistic interaction in terms of defeat or success affects the cortisol response at re-grouping after three weeks of isolation [36,37]. Those studies found that pigs which had previously experienced only defeats had a lower cortisol response than pigs with few or no defeats. This could explain the negative correlation between attacks initiated, attacks received and cortisol variation for primiparous sows, as they most likely experienced defeats when they were group-housed with older sows in the gestation pen and thus were more habituated to the outcome of the agonistic interaction at mixing post farrowing. The multiparous sows in the present study, however, were likely to have included some that had experienced defeats and some that had experienced success, and therefore exhibited different cortisol responses in the group housing. There was therefore no significant correlation between agonistic behaviour and cortisol variation among these sows. Furthermore, the positive correlation between scratch score and attacks received for multiparous sows could possibly be explained by the multiparous sows being more involved in agonistic interactions [36,38].

## Conclusions

Sows mixed three weeks post farrowing (W3 sows) showed lower cortisol variation than sows mixed one week after farrowing (W1 sows) and had a lower cortisol level when group-housed than when individually housed. This was not the case for the W1 or W2 sows. Overall, this suggests that mixing and group housing at three weeks post farrowing is a less stressful routine than group housing and mixing at one week post farrowing. Furthermore, when grouping sows, parity and whether a sow is a resident or intruder in the new environment could have an effect on agonistic behaviour at mixing. However, the effect of these different group housing and mixing routines on nursing-suckling interaction and, ultimately, the incidence of lactational oestrus and piglet mortality warrant further studies.
